# Radiomics-enhanced ^18^F-AV45 PET/MRI for integrative assessment and centiloid estimation of amyloid-β burden in Alzheimer’s disease

**DOI:** 10.1186/s41747-026-00766-3

**Published:** 2026-06-18

**Authors:** Zengbei Yuan, Jianzhou Zhang, Zirong Zhou, Xing Chen, Na Qi, Weilun Wang, Xinwei Cheng, Yingkang Lin, Jun Zhao

**Affiliations:** 1 Department of Nuclear Medicine, Shanghai East Hospital, School of Medicine, Tongji University, Shanghai, China; 2 Department of Rehabilitation, Guangdong Work Injury Rehabilitation Hospital, Guangzhou, China

**Keywords:** Alzheimer disease, Florbetapir, Machine learning, Positron emission tomography, Radiomics.

## Abstract

**Objective:**

Reliable assessment of cerebral amyloid-β (Aβ) deposition is essential for the diagnosis and management of Alzheimer's disease (AD). This study aimed to evaluate the feasibility of integrating radiomics-enhanced ^18^F-florbetapir positron emission tomography/magnetic resonance imaging (^18^F-AV45 PET/MRI) features for Aβ status evaluation and to further explore their potential for continuous Centiloid prediction in AD.

**Materials and methods:**

Ninety-four subjects who underwent (^18^F-AV45 PET/MRI (60 Aβ-positive, 34 Aβ-negative) were retrospectively included. Standardized uptake value ratio (SUVr) features were extracted from seven cortical regions (frontal, temporal, parietal, occipital, insular, cingulate, and white matter), and corresponding T1-weighted images' radiomics features were computed. Three feature sets (PET, radiomics, and combined) were analyzed using logistic regression (LR), k-nearest neighbor (kNN), and linear discriminant analysis (LDA) with 10-fold cross-validation. The best performing model was further interpreted using SHapley Additive exPlanations (SHAP) analysis. Additionally, Centiloid regression was performed using random forest, ElasticNet, and ExtraTrees regressors.

**Results:**

The combined feature achieved the best performance with the LR model, with area under the receiver operating characteristic curve = 0.9373, accuracy = 0.8723, F1-score = 0.898). SHAP analysis identified biologically meaningful features derived from both radiomics and PET modalities, showing clear inter-group separation. In Centiloid regression, the ExtraTrees model achieved strong agreement with measured values.

**Conclusion:**

This framework provides an interpretable and quantitative solution for amyloid evaluation, enabling both categorical Aβ status discrimination and continuous Centiloid estimation from routine PET/MRI data. This approach represents a proof-of-concept for supporting ^18^F-AV45 PET-based assessment in AD.

**Relevance statement:**

This study demonstrates that radiomics-enhanced PET/MR features can reliably predict both Aβ status and Centiloid values without specialized processing platforms, offering a clinically deployable, standardized, and interpretable approach to improve AD diagnosis and monitoring.

**Key Points:**

- Radiomics-enhanced ^18^F-AV45 PET/MRI enabled quantitative Aβ evaluation in AD.
- Radiomics-enhanced ^18^F-AV45 PET/MRI provided a noninvasive and interpretable assessment to improve clinical confidence.
- Radiomics-enhanced ^18^F-AV45 PET/MRI allowed Centiloid estimation without specialized platforms for wider clinical use.

**Graphical Abstract:**

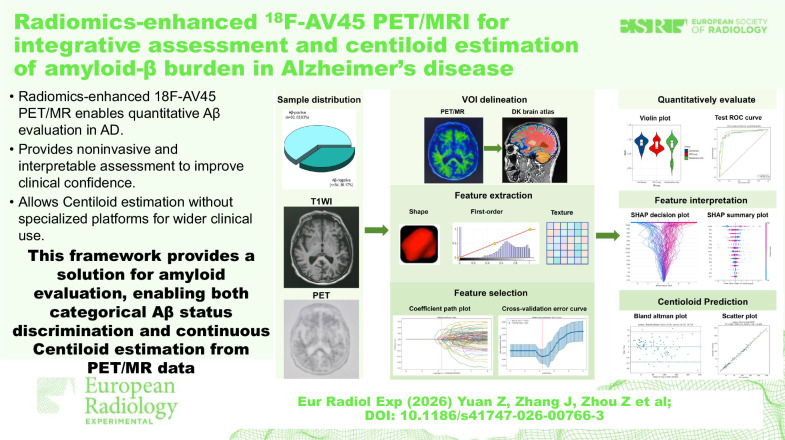

## Background

Alzheimer's disease (AD) is the most prevalent neurodegenerative dementia, accounting for the majority of cases in aging populations and imposing profound individual, caregiver, and societal burdens [1]. Clinically, AD spans a continuum from preclinical amyloidosis to prodromal mild cognitive impairment and dementia, with substantial heterogeneity in onset age, symptom profiles, and progression rates [2]. Conventional evaluation, such as history, neuropsychological testing, and routine MRI, can suggest neurodegeneration but cannot directly confirm the underlying proteinopathy. The shift toward biomarker-based diagnosis is exemplified by the amyloid-tau-neurodegeneration framework, in which “A” denotes amyloid-β (Aβ) deposition, “T” tau aggregation, and “ [N]” neurodegeneration [3]. In this context, reliable *in vivo* evidence of cerebral amyloidosis is pivotal for differential diagnosis, prognosis, clinical trial enrichment, and selection or monitoring of anti-amyloid therapies [4]. As disease-modifying strategies emerge, the clinical premium on accurate and reproducible amyloid assessment continues to rise.

Aβ deposition initially in neocortical association areas is a core histopathologic hallmark of AD. ^18^F-florbetapir positron emission tomography (^18^F-AV45 PET) visualizes fibrillar Aβ plaques and has become integral to adjudicating ambiguous cognitive syndromes, clarifying the etiology in atypical or mixed presentations, and supporting therapeutic decisions [5–7]. Positron emission tomography/magnetic resonance imaging (PET/MRI) enables joint assessment of molecular pathology and macrostructural context, improving anatomical localization and reader confidence [8]. In clinical practice, Aβ status discrimination on ^18^F-AV45 PET is primarily determined by visual reads, often aided by simple standardized uptake value ratio (SUVr)-based rules within predefined cortical regions. While these approaches are practical and widely adopted, their accuracy may decline in borderline or diffuse uptake patterns, under motion or partial-volume effects, and across different scanners or processing pipelines. Moreover, visual interpretation remains reader-dependent, and SUVr thresholds lack standardization, frequently leading to inconsistent classifications across sites. Although the Centiloid framework offers a harmonized quantitative scale, its reliance on dedicated software and non-integrated processing limits its accessibility in routine clinical workflows [9].

Radiomics and machine learning (ML) provide a complementary pathway for improving amyloid assessment [10]. Radiomic features are typically categorized into shape, first-order, and texture features. Shape features describe spatial extent and geometric properties of the region-of-interest itself; first-order features quantify the statistical distribution of voxel intensities; and texture features characterize spatial gray-level relationships, reflecting intra-regional heterogeneity and structural organization [11]. Radiomics quantifies high-dimensional descriptors of intensity, texture, and morphology from T1-weighted imaging (T1WI), potentially capturing subtle cortical alterations related to amyloid pathology that may not be apparent on visual inspection [12]. When integrated with regional SUVr, these radiomic features can assist clinicians in determining Aβ positivity more objectively, providing quantitative support to conventional visual or SUVr-based reads. Unlike Centiloid quantification, which requires specialized software, harmonized calibration, and dedicated processing infrastructure, radiomics-based approaches are easier to configure and can be implemented directly within standard radiology workflows. Furthermore, recent advances in interpretable ML allow transparent visualization of feature contributions, enabling clinicians to understand how radiomic characteristics influence model decisions. Together, these developments make radiomics-assisted SUVr analysis a practical and explainable tool for improving the reliability of amyloid evaluation in clinical settings [13].

Accordingly, this study retrospectively analyzed 94 subjects who underwent ^18^F-AV45 PET/MRI scanning to evaluate whether integrating regional PET SUVr with T1WI-based radiomic features could improve Aβ-positivity classification. Three feature sets (PET, radiomics, and combined) were constructed, and classic ML classifiers were trained using stratified 10-fold cross-validation. The best-performing combined model was further interpreted using SHapley Additive exPlanations (SHAP) analysis with constrained top-feature visualization to enhance interpretability. Additionally, a supplementary regression experiment was conducted on Centiloid values to assess the consistency and quantitative potential of the proposed framework.

## Methods

### Enrolled participants

This study included a total of 94 consecutive participants who underwent ^18^F-AV45 PET/MRI scanning between February 2023 and July 2025. All images were de-identified before analysis. Demographic and clinical characteristics of the study cohort are summarized in Table 1. Eligible participants had completed ^18^F-AV45 PET/MRI examinations during the study period and had complete demographic and clinical data. Exclusion criteria were: (1) history of stroke with focal neurological deficits; (2) presence of other neurological disorders that may cause brain dysfunction, including brain tumors, metabolic encephalopathy, encephalitis, multiple sclerosis, epilepsy, and traumatic brain injury; (3) presence of systemic diseases that may lead to cognitive impairment, such as liver dysfunction, renal dysfunction, thyroid abnormalities, severe anemia, folate or vitamin B12 deficiency, syphilis, human immunodeficiency virus infection, and substance or alcohol abuse; (4) presence of intellectual disability or neurodevelopmental disorders.

**Table 1:** Demographics of enrolled subjects

Characteristic	Total (*N*)	Aβ-positive	Aβ-negative	*p*-value
Number	94	60 (63.83%)	34 (36.17%)	
Age	69.98 ± 8.13	72.10 ± 7.00	66.24 ± 8.63	*p* < 0.05
Sex				0.325
M	34 (36.17%)	19 (31.67%)	15 (63.83%)	
F	60 (63.83%)	41 (68.33%)	19 (55.88%)	
Weight	62.11 ± 11.03	60.32 ± 9.70	65.26 ± 12.44	0.054
Height	162.65 ± 7.79	162.23 ± 7.21	163.38 ± 8.66	0.497
Centiloid	60.03 ± 61.86	90.27 ± 56.73	6.66 ± 20.90	*p* < 0.001

Data are frequencies with percentages in parentheses or means ± standard deviations

*Aβ* Amyloid-β

### Data acquisition

Each participant received an intravenous injection of ^18^F-AV45 and underwent brain PET/MRI scanning approximately 60 min after injection using a 3-T uPMR790 TOF scanner (United Imaging). The total acquisition time was about 30 min [14]. Participants were positioned supine, with cotton earplugs to reduce noise and foam pads between the head and the coil to minimize motion, and were instructed to remain still and keep their eyes closed throughout the scan. PET images were reconstructed with a three-dimensional ordered-subsets expectation maximization algorithm, using two iterations and 20 subsets. T1WI parameters were as follows: slice thickness 1.0 mm; matrix 256 × 256; field of view 230 × 230 mm²; repetition time 2,300 ms; echo time 3 ms; and flip angle 10°.

### Participant grouping

The PET/MRI reports were determined by two experienced nuclear medicine physicians. An initial read was performed by a physician with 10 years of diagnostic experience, and independently reviewed by a physician with 25 years of experience. Any discrepancies were resolved by consensus. Image assessment followed the 2011 National Institute on Aging-Alzheimer’s Association criteria for probable AD [15] and integrated PET imaging characteristics with essential clinical information. Both readers were blinded to quantitative SUVr and Centiloid results, as well as to the final diagnostic grouping. Based on the consensus interpretation of the amyloid-tau-neurodegeneration, participants were classified into Aβ-positive (*n* = 60) and Aβ-negative (*n* = 34) groups. Centiloid values were available for all participants and were used only for supplementary analyses [16].

### Image analysis

For each participant, the corresponding T1WI was preprocessed using the United Imaging Healthcare uAI Research Portal platform [17]. The preprocessing pipeline included image filtering, tissue segmentation, image registration, and intensity normalization. Specifically, the uAI Research Portal platform applied noise filtering and intensity correction, removed non-brain tissues, aligned images to a standardized anatomical template, and the T1WI used for radiomic analysis was acquired with isotropic 1 × 1 × 1 mm³ voxels and was not resampled to an anisotropic resolution during preprocessing. These steps ensured that all T1WI data were standardized and ready for subsequent radiomics feature extraction and ML analysis.

For PET images, the uAI Research Portal platform automatically segmented the brain into seven regions, including the frontal lobe, temporal lobe, parietal lobe, occipital lobe, insula, cingulate gyrus, and white matter. SUV maps were computed from the raw PET data and normalized by the injected dose and body weight. Brain parcellation was performed using the Desikan–Killiany atlas [18], yielding 93 regions of interest. Regional SUVmean values were extracted, and SUVRs were computed using the whole cerebellum as the reference region. Partial volume correction was not applied. To account for head motion, reconstructed PET images were rigidly coregistered to the subject’s T1WI prior to quantitative analysis. Critically, SUVr-to-centiloid conversion was implemented in accordance with Section 2.2.2.3.2 of the Klunk et al guidelines [9], calibration of another tracer or method for general use, using the exact published transformation parameters derived from the ¹¹C‑Pittsburgh Compound‑B, ¹⁸F‑Florbetaben, ¹⁸F‑Florbetapir, and ¹⁸F‑Flutemetamol datasets hosted on the Global Alzheimer’s Association Interactive Network website (https://gaain.org) [19].

### Feature extraction and selection

For each enrolled patient, each brain region radiomics feature extraction were conducted on the uAI Research Portal. All radiomics feature extraction procedures were performed in strict accordance with the Imaging Biomarker Standardization Initiative guidelines. A total of 2,264 quantitative features were extracted independently from T1WI modalities, encompassing 450 first-order features, 14 shape features, and 1,800 texture features. These features comprehensively captured the voxel intensity distribution, morphological characteristics, and multi-scale spatial heterogeneity of the brain region.

Given the high dimensionality of radiomics data, a two-step feature reduction strategy was adopted to mitigate overfitting and improve model generalizability. In the first stage, the full feature set (2,264 features per modality) was subjected to least absolute shrinkage and selection operator (LASSO) regression separately for each imaging subset, including each brain region. LASSO regularization was performed using a 5-fold cross-validation scheme to determine the optimal penalization parameter (λ = 0.1), with the mean squared error serving as the objective function. The L1 constraint progressively shrank the coefficients of non-informative or redundant features toward zero, thereby retaining only those with the strongest predictive contribution. As a result, a compact yet informative subset of features was preserved for each region combination. After feature selection, 20, 20, 20, 18, 19, 20, and 20 radiomics features were retained from the frontal, temporal, parietal, occipital, insular, cingulate, and white matter regions, respectively, resulting in a total of 137 radiomics features that collectively constituted the T1WI-radiomics set for subsequent model development.

These selected features were subsequently standardized (*z*-score normalization) and forwarded to downstream model development pipelines. The LASSO-based dimensionality reduction not only improved computational efficiency but also facilitated biological interpretability by emphasizing robust, reproducible, and nonredundant imaging biomarkers across both brain regions.

### Statistical analysis

Statistical analysis was performed using SPSS software (version 21.0; IBM Corp.). Continuous variables were tested for normality using the Shapiro–Wilk test. Variables conforming to a normal distribution were expressed as mean ± standard deviation and compared between groups using the independent-samples *t*-test; otherwise, data were expressed as median and compared using the Mann–Whitney *U*-test. Categorical variables were summarized as counts and percentages, and group differences were assessed using the chi-square test. All statistical tests were two-sided, and a *p*-value < 0.05 was considered statistically significant.

## Results

### Overall framework for Aβ assessment using radiomics-enhanced ^18^F-AV45 PET/MRI

**Fig. 1:**
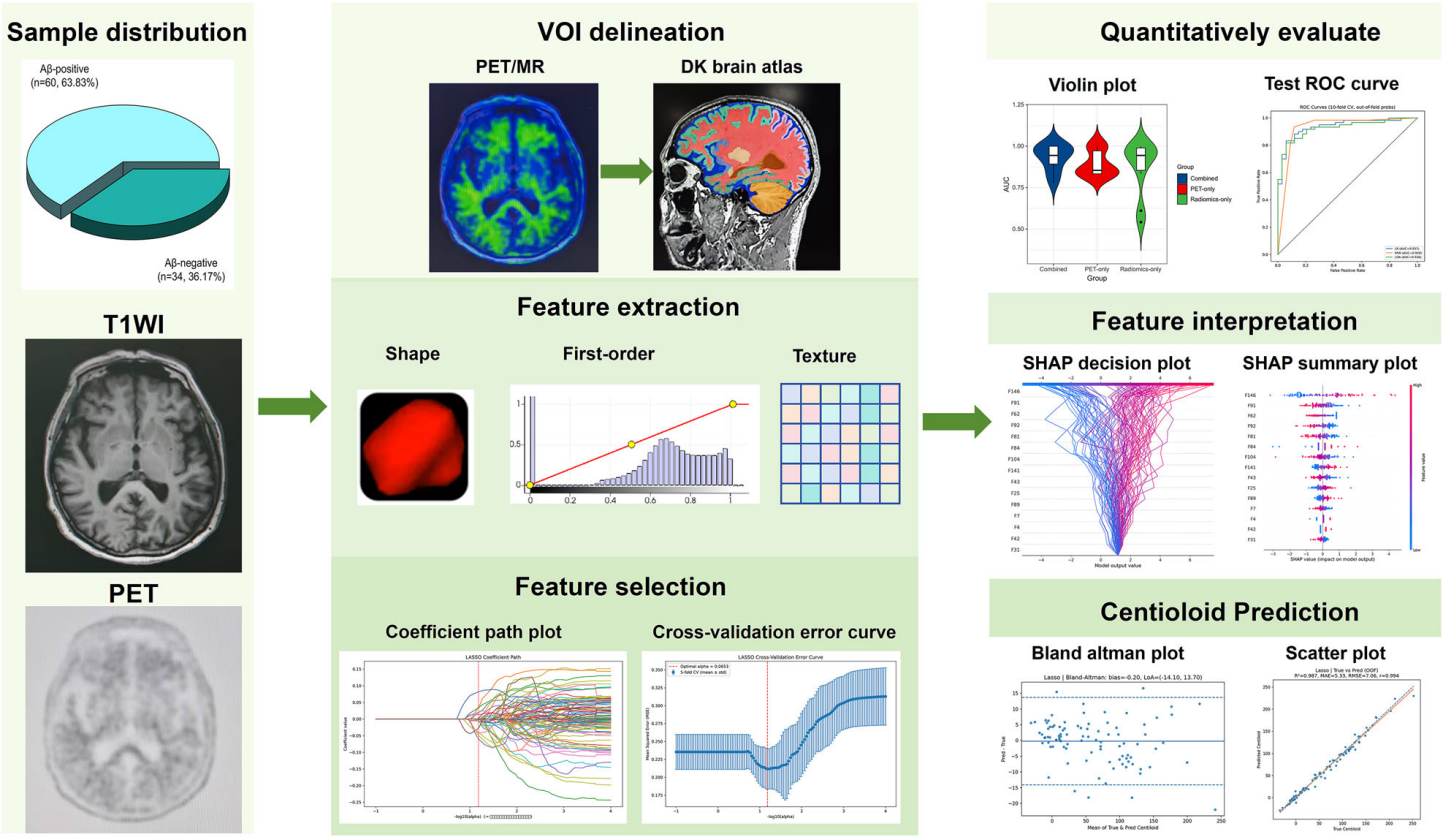
The overall framework of the proposed assessment and estimation model

To evaluate cerebral Aβ deposition status, we established a multiparametric ^18^F-AV45 PET/MRI-based framework integrating regional SUVr and T1WI-radiomics features with ML modeling (Fig. 1). The subjects who underwent PET/MRI were retrospectively analyzed. Cortical volumes of interest were segmented on T1WI according to the standardized Desikan–Killiany brain atlas [20]. For each predefined region, high-dimensional radiomic features capturing textural and morphological characteristics were extracted from T1WI [21], while regional SUVr were calculated from the PET image registered to the same atlas. To reduce dimensionality and minimize overfitting, the LASSO method was applied to select the most informative radiomic features [22, 23]. Subsequently, classic ML classifiers were trained on PET, radiomics, and combined feature sets using stratified 10-fold cross-validation. Model discrimination and calibration were comprehensively evaluated, and interpretability was further assessed using SHAP-based analysis to visualize the key imaging features contributing to Aβ status classification [24].

### Predictive performance of models

**Fig. 2:**
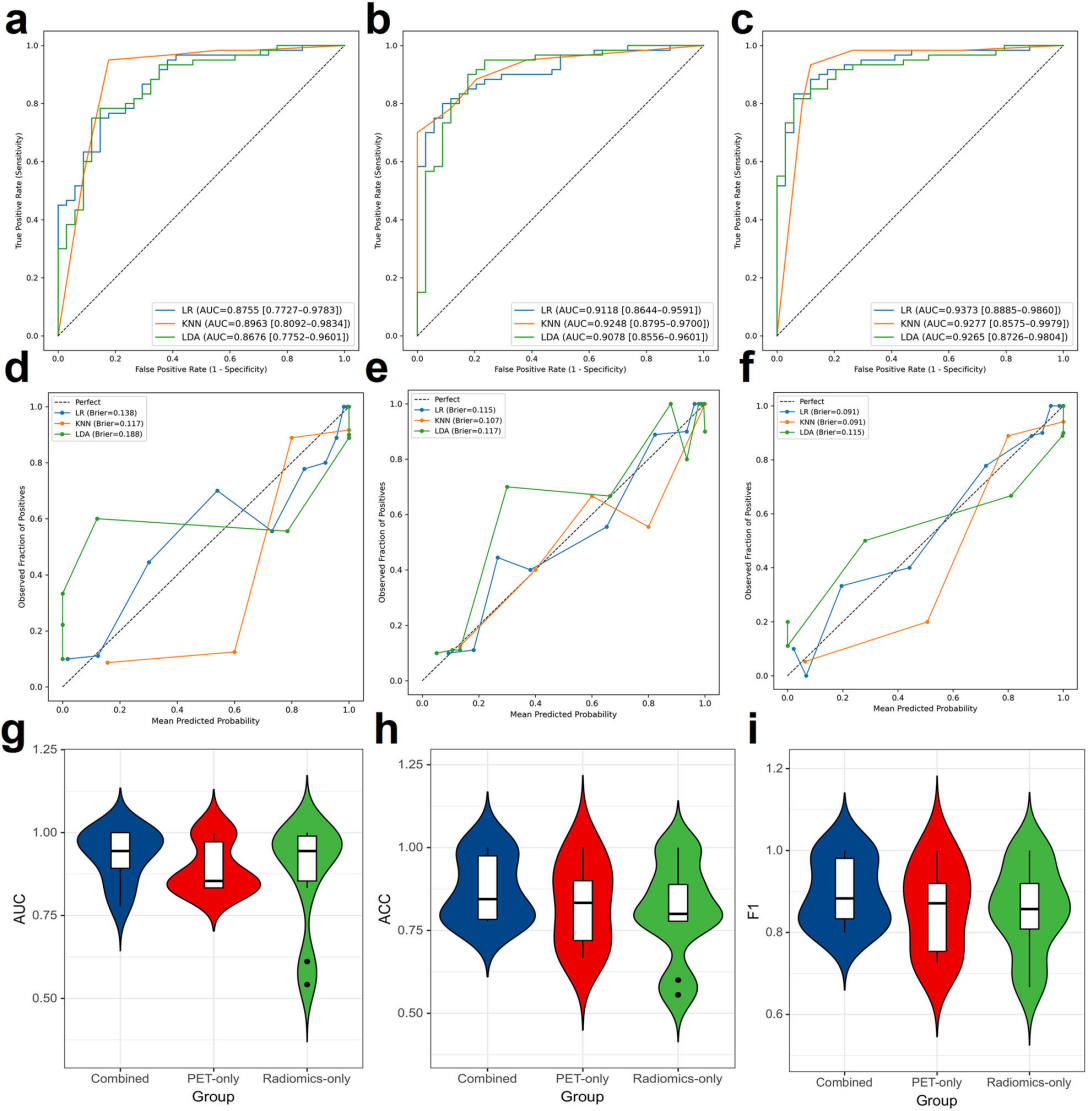
Performance of ML models across different feature sets. AUROC curves of LR, kNN, and LDA based on (**a**) radiomics, (**b**) PET, and (**c**) combined feature sets. Calibration curves of LR, kNN, and LDA based on (**d**) radiomics, (**e**) PET, and (**f**) combined feature sets. Violin plots showing cross-validated AUROC (95% confidence interval) (**g**), accuracy (**h**), and F1-score (**i**) distributions across radiomics, PET, and combined groups. AUROC, Area under the receiver operating characteristic curve; kNN, k-nearest neighbor; LDA, Linear discriminant analysis; LR, Logistic regression; PET, Positron emission tomography/magnetic resonance imaging

Three conventional ML classifiers, including logistic regression (LR), k-nearest neighbor (kNN), and linear discriminant analysis (LDA), implemented in Python using scikit-learn, were applied to three different feature sets (PET, radiomics, and combined). All features were standardized using StandardScaler prior to modeling. No class imbalance handling (*e.g*., sample weighting, Synthetic Minority Over‑sampling Technique, or downsampling) was applied; models were trained on the original class distribution. Model performance was estimated using stratified 10-fold cross-validation with shuffling (random_state = 42). All modeling steps, including feature standardization and classifier training, were confined within the cross-validation loop to prevent data leakage. However, feature selection via LASSO was performed once on the full dataset prior to cross-validation, and the resulting feature set remained fixed across all folds. As summarized in Table 2 and Fig. 2, all models achieved satisfactory classification performance, while the combined feature set consistently outperformed both PET and radiomic groups across all classifiers. Specifically, the combined feature set yielded the highest area under the receiver operating characteristic curve (AUROC), accuracy, and F1-score, suggesting that integrating PET-derived metabolic information with MRI-based radiomic descriptors provides complementary diagnostic value. Among the three classifiers, the LR model achieved the best overall performance within the combined feature set (AUROC 0.937, accuracy 0.8723, and F1 0.898), outperforming kNN (0.928, 0.894, and 0.922), and LDA (0.927, 0.8511, and 0.881, respectively). These results confirm that the LR model with combined features achieves the most reliable and discriminative performance, and it was therefore selected for subsequent SHAP-based interpretability analysis.

**Table 2:** Predictive performance of models

Group	Model	AUC	ACC	SEN	SPE	F1
PET	KNN	0.9248	0.8511	0.8833	0.7941	0.8833
	LDA	0.9078	0.8404	0.8333	0.8529	0.8696
	LR	0.9118	0.8298	0.8333	0.8235	0.8621
Radiomics	KNN	0.8963	0.8404	0.9667	0.6176	0.8855
	LDA	0.8676	0.7872	0.8	0.7647	0.8276
	LR	0.8755	0.8085	0.8833	0.6765	0.8548
Combined	KNN	0.9277	0.8936	0.9833	0.7353	0.9219
	LDA	0.9265	0.8511	0.8667	0.8235	0.8814
	LR	0.9373	0.8723	0.8833	0.8529	0.8983

*kNN* k-nearest neighbor, *LDA* Linear discriminant analysis, *LR* Logistic regression

Due to the significant age difference between the Aβ-positive and Aβ-negative groups in our cohort, we conducted age-related control analyses. These results confirmed that age alone provides limited discriminative value for classifying Aβ status, and that including it in the multimodal model did not improve performance. This suggests that our imaging biomarkers capture Aβ-related pathology beyond chronological age. Full details are provided in the supplementary materials (Supplementary Tables S1 and Table S2).

### Model explanation with SHAP

**Fig. 3:**
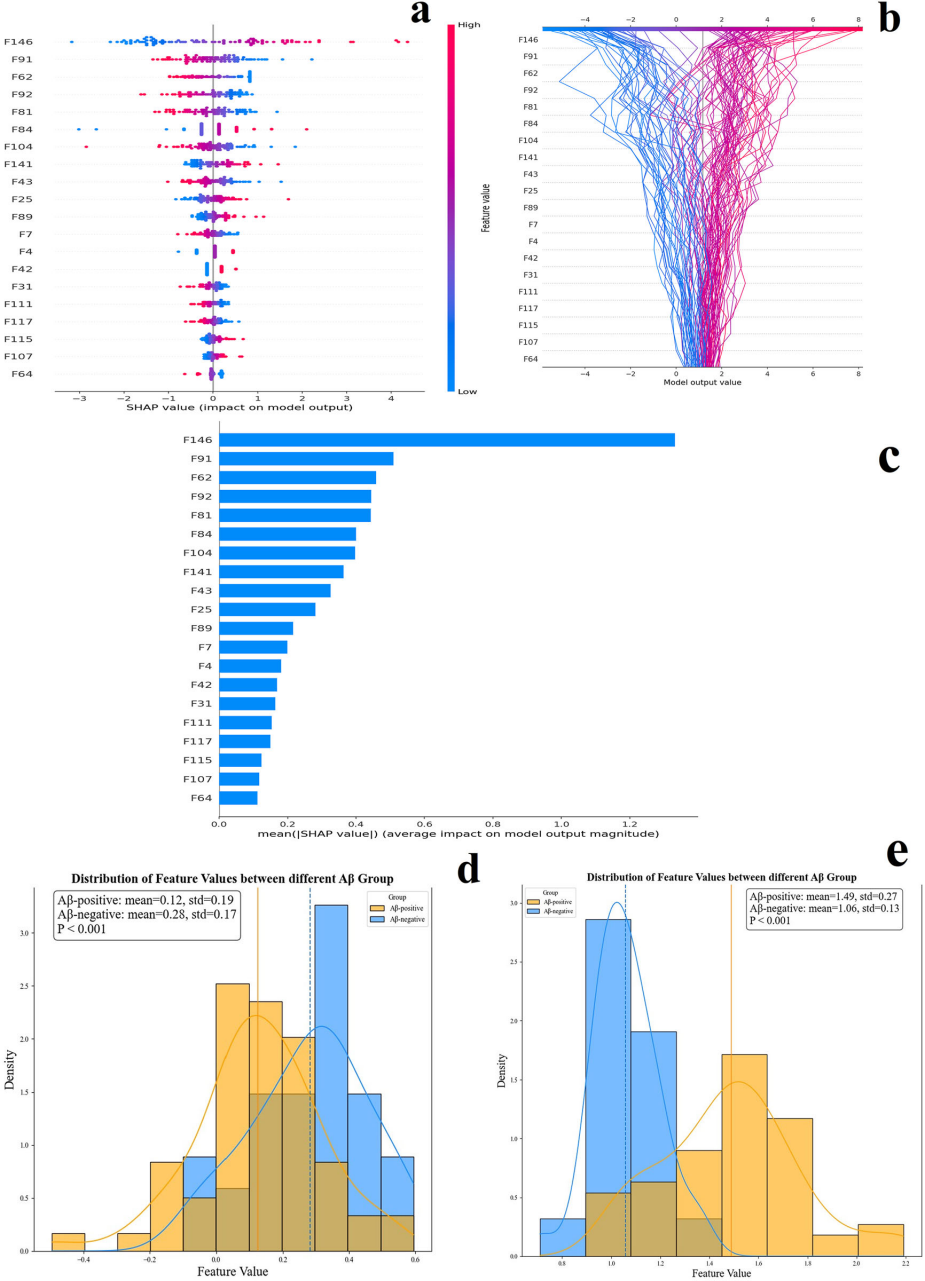
SHAP-based interpretability and key feature distributions of the LR. SHAP summary (**a**), decision (**b**), and bar (**c**) plots showing the most influential features. Histograms with kernel density estimation illustrating group-wise distributions of top SHAP-ranked features: F91 (**d**) and F146 (**e**) between Aβ-positive and Aβ-negative groups. LR, Logistic regression; SHAP, SHapley Additive exPlanations

To enhance the interpretability of the LR model trained on the combined feature set, SHAP analysis was performed to quantify and visualize the contribution of each feature to the model output. As shown in Fig. 3a, the SHAP summary plot demonstrated that features R-parietal-SUVr (F146), R-cingulate-Laplaciansharpening-first-order-Skewness (F91), and L-frontal-laplaciansharpening-first-order-Minimum (F62) exerted the strongest influence on model predictions, with red and blue points representing high and low feature values, respectively. Higher SHAP values were associated with a greater likelihood of Aβ positivity. The SHAP decision plot (Fig. 3b) illustrated the cumulative impact of these features across all subjects, showing that elevated values of F146 and F91 consistently pushed the model output toward the positive class. The SHAP bar plot (Fig. 3c) ranked the mean absolute SHAP values, with F146 contributing most substantially (> 1.2), followed by F91 and F62, highlighting a compact yet highly informative subset of features driving model behavior.

To further validate these interpretability findings, the two most influential features identified by F91 and F146 were analyzed for group-level distribution. As shown in Fig. 3d, e, histograms with kernel density estimation revealed distinct between-group separations. Specifically, F91 (Fig. 3d) exhibited lower mean values in the Aβ-positive group (0.12 ± 0.19) than in the Aβ-negative group (0.28 ± 0.17, *p* < 0.001), whereas F146 (Fig. 3e) showed the opposite trend, with significantly higher values in the Aβ-positive group (1.49 ± 0.27) compared with the Aβ-negative group (1.06 ± 0.13, *p* < 0.001). These results confirm that SHAP-identified high-impact features not only drive model prediction but also reflect biologically meaningful differences between clinical groups.

### Quantitative prediction of Centiloid

To further examine the quantitative applicability of the proposed framework, regression experiments were conducted to predict Centiloid values using three ML models (ElasticNet, ExtraTrees, and random forest) trained on the combined feature set. As summarized in Table 3, all models demonstrated strong predictive performance, with high coefficients of determination and low prediction errors. Among them, the ExtraTrees model achieved the most favorable results, yielding a mean absolute error of 6.461, a root mean square error of 10.722, and an *R*² value of 0.970. These metrics represent the mean values obtained across 10-fold cross-validation runs. These results indicate that the established feature representation retains stable predictive power in continuous Centiloid value estimation, supporting its potential utility in quantitative imaging analysis.

**Table 3:** Performance of ML models in Centiloid estimation

Model	MAE	RMSE	*R*^2^
ElasticNet	6.544	9.7814	0.9751
ExtraTrees	6.4609	10.7215	0.9701
LASSO	5.3255	7.0559	0.9871

*MAE* Mean absolute error, *RMSE* Root mean square error

**Fig. 4:**
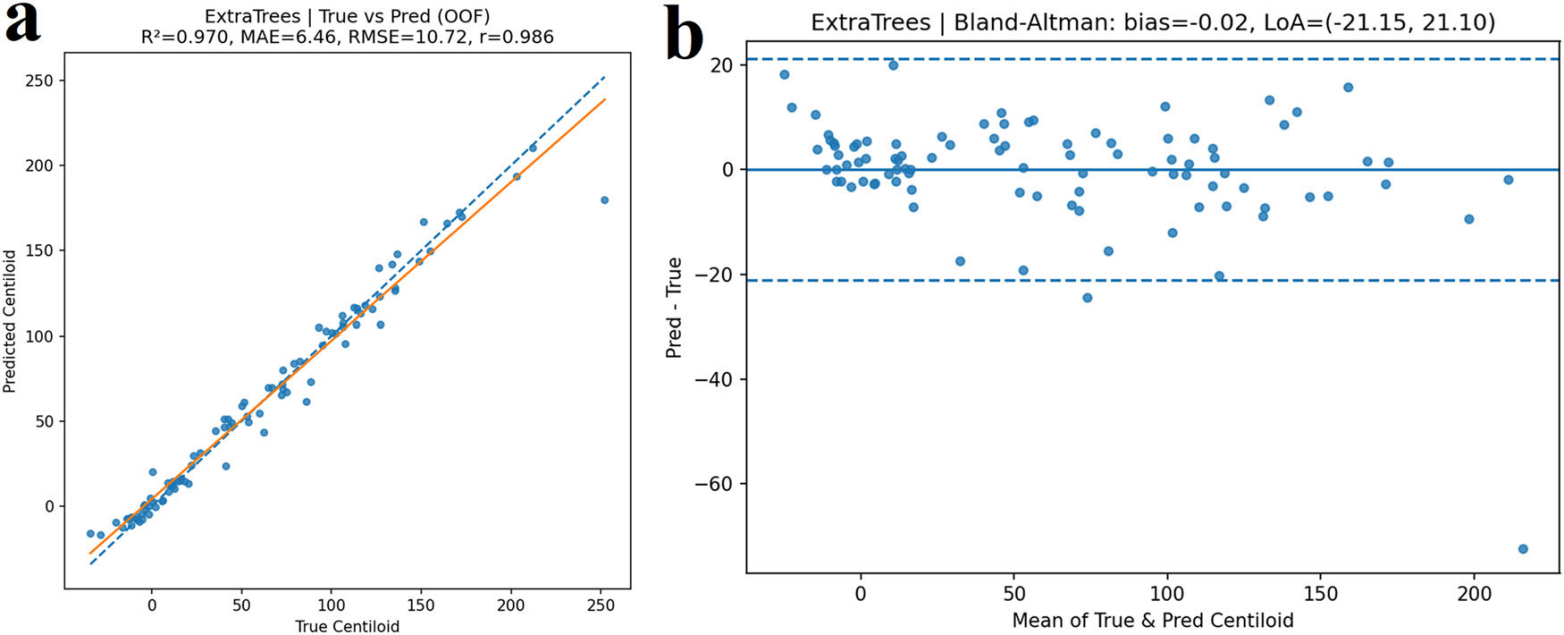
Regression performance and agreement analysis of the ExtraTrees: (**a**) scatter plot; (**b**) Bland–Altman plot

Given the superior performance of the ExtraTrees model, its regression results were further visualized to illustrate the relationship between predicted and measured Centiloid values. As shown in Fig. 4a, the scatter plot demonstrates a strong linear correlation between predicted and true values, with the regression line nearly overlapping the identity line, indicating minimal deviation across the full Centiloid range. The Bland Altman plot (Fig. 4b) further confirms high agreement between predicted and observed values, with limits of agreement between -21.15 and 21.10. Most data points fall within the 95% confidence interval, suggesting stable and reliable regression performance of the ExtraTrees.

## Discussion

In this study, we developed and validated a radiomics-enhanced ^18^F-AV45 PET/MRI framework for assessing cerebral Aβ deposition. The results demonstrated that the combined feature representation achieved high classification accuracy for Aβ positivity and maintained robust performance in continuous Centiloid value prediction [25, 26]. Furthermore, SHAP-based interpretability analyses identified biologically plausible imaging features driving model decisions, thereby enhancing both the transparency and the potential clinical applicability of the proposed approach. Critically, our approach is designed to address a real-world clinical need: enabling quantitative amyloid assessment in routine care settings where dedicated Aβ PET quantification software or standardized Centiloid processing pipelines are unavailable or impractical to implement. By leveraging only routinely acquired PET/MRI and without requiring external tools, manual segmentation, or specialized expertise, this framework offers a practical, integrable solution for noninvasive, quantitative, and explainable amyloid evaluation. As such, it holds potential value for early detection, risk stratification, and longitudinal monitoring in AD, particularly in resource-limited or community-based healthcare environments.

The radiomics-augmented PET/MRI framework provided complementary molecular and structural information, allowing for a more comprehensive assessment of amyloid-related brain alterations [27]. The SUVr feature captured regional tracer uptake indicative of Aβ burden, whereas radiomic descriptors derived from T1WI quantified subtle textural and morphological variations that may accompany amyloid accumulation. The synergistic integration of these complementary modalities likely enhanced model discriminative power, as reflected by the superior performance of the combined feature set relative to single-modality inputs. These results are consistent with prior evidence that multiparametric imaging biomarkers offer a more robust depiction of neurodegenerative processes than any individual modality alone. Among the tested classifiers, LR achieved the most balanced and stable performance across evaluation metrics, yielding an AUROC of 0.937 and an accuracy of 0.872 for Aβ positivity assessment. Its linear formulation and sparsity-promoting parameterization also facilitated downstream interpretability analysis. The SHAP results highlighted several high-impact features (F91, F146, and F62) that exerted a strong influence on model outputs and exhibited consistent between-group differences between Aβ-positive and Aβ-negative participants. Both features (F91, F62) were significantly lower in the Aβ-positive group compared to the Aβ-negative group, suggesting that early amyloid pathology is associated with measurable alterations in gray matter intensity distribution within these vulnerable regions. The posterior cingulate cortex, a key hub of the default mode network, is known to undergo early microstructural disruption in AD; reduced skewness may reflect a loss of tissue heterogeneity due to neuronal degeneration or gliosis [28]. Similarly, decreased minimum intensity in the left frontal lobe may indicate subtle cortical thinning or reduced gray–white matter contrast, consistent with early neurodegenerative changes. These observations align with prior radiomics studies by Li et al and Gao et al [29, 30], who reported that first-order intensity features from the cingulate and frontal regions are sensitive to AD-related pathological processes, further supporting the biological plausibility of our imaging biomarkers. The concordance between SHAP-derived importance and empirical group contrasts underscores the biological plausibility and clinical relevance of the identified predictors.

Beyond Aβ status discrimination, the framework was further extended to predict continuous Centiloid values, which offer a standardized quantitative index of amyloid burden [31]. The regression experiments demonstrated excellent performance, particularly for the ExtraTrees model, with close agreement between predicted and measured Centiloid values [32, 33]. The Bland–Altman analysis revealed minimal bias and narrow limits of agreement, confirming the quantitative reliability of the model’s predictions. Importantly, although the Centiloid scale provides a widely recognized standard for cross-study amyloid quantification, its routine clinical adoption remains limited by reliance on specialized software platforms, standardized spatial normalization templates, and expert-dependent processing pipelines. In contrast, the proposed radiomics-enhanced PET/MRI framework leverages routinely acquired clinical scans, without requiring external tools or manual intervention, to enable data-driven estimation of Centiloid-equivalent values. While not intended to replace certified Centiloid protocols [34], this approach offers a practical and accessible pathway toward quantitative amyloid assessment in resource-constrained or workflow-integrated clinical settings.

The proposed framework offers several potential clinical benefits. First, it provides an interpretable and automated approach for assessing Aβ burden that may complement visual reads, particularly in borderline or equivocal cases. Second, by jointly quantifying structural and metabolic characteristics, the model can facilitate longitudinal monitoring of disease progression and therapeutic response. Third, by enabling data-driven estimation of Centiloid values directly from routinely acquired PET/MRI data, the framework provides a practical and accessible alternative for standardized amyloid quantification without reliance on dedicated processing platforms. Moreover, the incorporation of SHAP-based interpretability ensures that model decisions remain transparent and clinically explainable, an essential prerequisite for clinical and regulatory translation [35]. Despite these strengths, several limitations should be acknowledged. The retrospective, single-center design and modest sample size may limit the generalizability of our model’s findings, which could be influenced by site-specific biases or demographic imbalances. External validation in larger, multicenter, prospective cohorts is essential to assess generalizability. Furthermore, LASSO feature selection was performed on the entire dataset before cross-validation, meaning that the resulting feature set remained fixed across all folds. While this strategy improved feature stability, given our small sample size, the feature space was informed by the entire cohort, which could have led to optimistic bias in performance estimates. Future studies with larger samples should implement fully nested pipelines to eliminate this source of bias. Furthermore, while expert visual reads were performed blind to quantitative outputs, the Aβ status labels and SUVr features both originated from the same PET acquisition. While this shared data source was unavoidable in the absence of orthogonal reference standards, it reduces the independence between the ground truth and the model inputs, which is a known limitation of single-modality imaging biomarker studies. Moreover, only conventional ML classifiers were explored. Future studies may investigate deep learning or hybrid fusion strategies to capture higher-order feature interactions. Finally, integrating additional imaging or non-imaging biomarkers, such as tau PET or genetic risk factors (*e.g.*, apolipoprotein E), may further enhance predictive robustness and biological interpretability.

In conclusion, this study established an interpretable ML framework based on radiomics-enhanced ^18^F-AV45 PET/MRI imaging for assessing cerebral Aβ burden. The proposed model demonstrated high diagnostic accuracy for Aβ status discrimination and robust performance in continuous Centiloid estimation. SHAP-based interpretability further revealed biologically plausible and clinically meaningful imaging features. Collectively, these findings highlight the potential of radiomics-guided PET/MRI modeling as a noninvasive, quantitative, and explainable tool for amyloid evaluation, offering practical value for early diagnosis and longitudinal monitoring in AD.

## Supplementary information

**Additional File 1: Table S1.** Predictive performance of age models. **Table S2.** Predictive performance of our models without age.

## Data Availability

The datasets generated during or analyzed during the current study are available from the corresponding author on reasonable request.

## Abbreviations

^18^F-AV45 PET: ^18^F-florbetapir positron emission tomography
AD: Alzheimer’s disease
AUROC: Area under the receiver operating characteristic curve
Aβ: Amyloid-β
F146: R-parietal-SUVr
F62: L-frontal-laplaciansharpening-first-order-Minimum
F91: R-cingulate-Laplaciansharpening-first-order-Skewness
kNN: k-nearest neighbor
LASSO: Least absolute shrinkage and selection operator
LDA: Linear discriminant analysis
LR: Logistic regression
ML: Machine learning
MRI: Magnetic resonance imaging
PET: Positron emission tomography
SHAP: SHapley Additive exPlanations
SUVr: Standardized uptake value ratio
T1WI: T1-weighted imaging
