# Supplementary material for: Radiomics-enhanced 18F-AV45 PET/MRI for integrative assessment and centiloid estimation of amyloid-β burden in Alzheimer’s disease
Source: Eur Radiol Exp. 2026 Jun 18;10:92. doi: 10.1186/s41747-026-00766-3 (PMC13280297; doi:10.1186/s41747-026-00766-3)
Supplement: Supplementary file 1 — Additional File 1: Table S1. Predictive performance of age models. Table S2. Predictive performance of our models without age. [file 41747_2026_766_MOESM1_ESM.pdf]

Radiomics-enhanced 18 F-AV45 PET/MRI for integrative assessment and Centiloid estimation of amyloid-β burden in Alzheimer disease

ELECTRONIC SUPPLEMENTARY MATERIAL

Supplementary table S1: Predictive performance of age models

Model	AUC	ACC	SEN	SPE	F1
LDA	0.6963	0.6702	0.8833	0.2941	0.7737
LR	0.6949	0.6489	0.85	0.2941	0.7556
KNN	0.6297	0.6383	0.8167	0.3235	0.7424

kNN k-nearest neighbor, *LDA* Linear discriminant analysis, LR Logistic regression.

Supplementary table S2: Predictive performance of our models without age

Model	AUC	ACC	SEN	SPE	F1
LR	0.9373	0.8404	0.8333	0.8529	0.8696
KNN	0.9167	0.8936	0.95	0.7941	0.9194
LDA	0.9157	0.8723	0.8833	0.8529	0.8983

kNN k-nearest neighbor, *LDA* Linear discriminant analysis, LR Logistic regression.
